# The oral-gut axis in chronic atrophic gastritis: current perspectives and integrated strategies

**DOI:** 10.3389/fimmu.2025.1699501

**Published:** 2026-01-08

**Authors:** Tianyue Zha, Yonggang Ding, Xingli Xu, Yifan Zhang, Jinwei Guo, Huinan Ge, Luzhou Xu

**Affiliations:** 1 Suzhou TCM Hospital Affiliated to Nanjing University of Chinese Medicine, Suzhou, China; 2 Xiyuan Hospital Suzhou of China Academy of Chinese Medical Sciences, Suzhou, China; 3 Department of Coloproctology, Yueyang Hospital of Integrated Traditional Chinese and Western Medicine, Shanghai University of Traditional Chinese Medicine, Shanghai, China; 4 Jiangsu Province Hospital of TCM Affiliated Hospital of Nanjing University of Chinese Medicine, Nanjing, China

**Keywords:** chronic atrophic gastritis, *Helicobacter pylori*, immune responses, intestinal metaplasia, microbiome dysbiosis, oral-gut axis, precision management

## Abstract

Chronic atrophic gastritis (CAG) is a key precursor to gastric cancer, characterized by progressive mucosal atrophy, inflammation, and microbial dysbiosis. The Correa cascade model highlights *Helicobacter pylori* as a primary driver, progressing from gastritis to atrophy, intestinal metaplasia (IM), dysplasia, and malignancy. However, 20%–30% of CAG cases lack *H. pylori* involvement, emphasizing the roles of non-*H. pylori* microbial dysbiosis, environmental factors, and the oral-gut axis in disease progression. Oral microbes, such as Porphyromonas gingivalis, translocate to the stomach, amplifying inflammation through NF-κB and Wnt/β-catenin pathways and altering metabolites like short-chain fatty acids and trimethylamine N-oxide. Pro-inflammatory cytokines, including IL-1β, IL-6, and IL-17, alongside Th17-driven immune dysregulation, further accelerate carcinogenesis. This perspective integrates multi-omics data to elucidate microbiome shifts, metabolic changes, and immune responses across CAG subtypes. Advanced diagnostics, such as endoscopic imaging, serum biomarkers, and oral microbiota profiling, enable precise risk stratification. Management strategies extend beyond *H. pylori* eradication to include probiotics, fecal microbiota transplantation, periodontal interventions, and herbal compounds, targeting the oral-gut axis to restore microbial balance and halt carcinogenesis. This framework offers novel avenues for prevention and therapy in high-burden regions.

## Introduction

Chronic atrophic gastritis (CAG) is a chronic inflammatory condition strongly linked to gastric cancer, contributing to a rising global disease burden, with prevalence rates of 5%–28% in high-risk regions (1–3). The Correa cascade model outlines the carcinogenic progression from *Helicobacter pylori* infection to gastritis, atrophy, intestinal metaplasia (IM), dysplasia, and gastric cancer (4, 5). Cytokines, including IL-1β and IL-6, drive chronic inflammation through nuclear factor-kappa B (NF-κB) and mitogen-activated protein kinase (MAPK) pathways (6). In gastric ulcers associated with CAG, caspase-mediated apoptosis, primarily through the mitochondrial pathway, causes extensive loss of epithelial cells, disruption of tight junctions, mucosal thinning, and glandular reduction, thereby compromising mucosal integrity (7); these ulcers and atrophic changes predominantly affect the antral region and antrum-corpus junction, mirroring the typical distribution of *H. pylori*-related lesions. *H. pylori*-positive patients exhibit a 2.4-fold higher CAG prevalence compared to *H. pylori*-negative individuals (2). However, 20%–30% of CAG cases lack *H. pylori* infection (8), with gastric mucosal dysbiosis—marked by reduced Lactobacillus and enriched Prevotella—activating toll-like receptor pathways, sustaining inflammation, and independently promoting carcinogenesis (9, 10). Microbial metabolites, such as diminished short-chain fatty acids (SCFAs) and accumulated secondary bile acids, further drive IM and dysplasia via Wnt/β-catenin signaling (11, 12). Metagenomic analyses reveal that microbiota diversity alterations impair mucosal barriers and amplify inflammation, limitations not fully addressed by traditional models (9).

**Figure 1:**
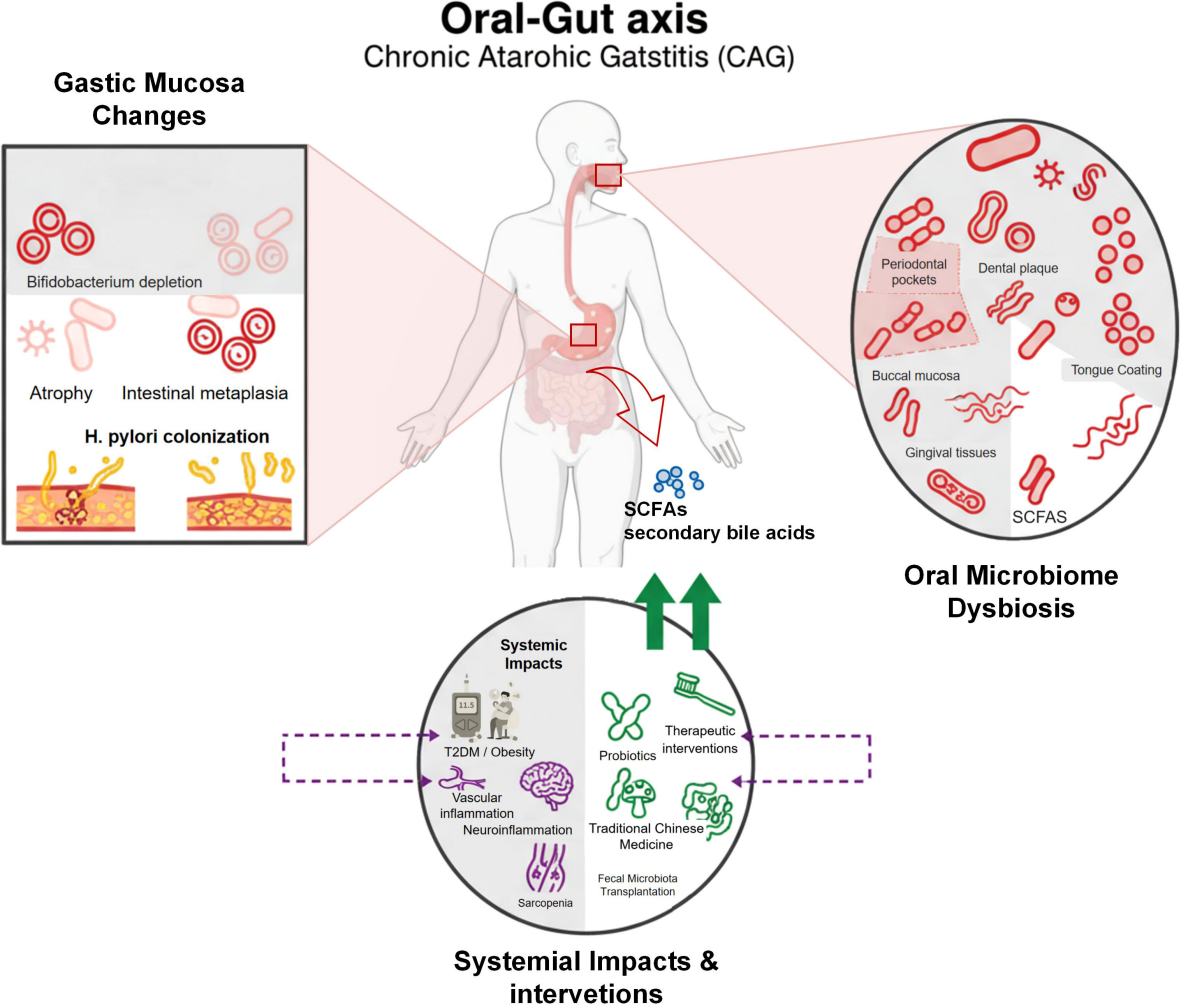
Oral-gut axis in chronic atrophic gastritis. This schematic illustrates the oral-gut axis in chronic atrophic gastritis (CAG). The right panel highlights oral microbiome dysbiosis in periodontal pockets, dental plaque, tongue coating, buccal mucosa, and gingival tissue, featuring microbes like Porphyromonas gingivalis, Fusobacterium nucleatum, *Helicobacter pylori*, *Candida albicans*, and *Treponema denticola*. The left panel shows gastric mucosa changes, including Streptococcus enrichment, Bifidobacterium depletion, *H. pylori* colonization, and precancerous atrophy and intestinal metaplasia. The bottom panel links systemic effects (e.g., T2DM, neuroinflammation, sarcopenia) to dysbiosis and outlines interventions—oral hygiene, probiotics (*Lactobacillus reuteri*), traditional Chinese medicine, and fecal microbiota transplantation—modulating the oral-gut axis.

Recent research highlights the oral-gut axis as a key contributor to CAG pathogenesis. Oral microbes, such as Porphyromonas gingivalis, translocate to the stomach, disrupting epithelial barriers and increasing mucosal permeability (13, 14). **Figure 1** illustrates the integrated role of the oral-gut axis in CAG, highlighting microbial dysbiosis, translocation pathways, and therapeutic interventions. Metabolites from gut microbiota, including lipopolysaccharide (LPS) from Gram-negative bacteria and trimethylamine N-oxide (TMAO) derived from trimethylamine, synergize to amplify inflammation through NF-κB activation, elevating CAG progression risk (15). *H. pylori* in periodontal tissues exacerbates inflammation via cross-organ migration or systemic immune responses. Aberrant Th17 cell activation, involving IL-17, IL-21, and IL-23 secretion, contributes to periodontal and gastric immunopathology, underscoring bidirectional microbial-immune interactions (16).

This perspective focuses on the oral microecosystem, encompassing periodontal pockets, dental plaque, tongue coating, buccal mucosa, and gingival tissues, which influences CAG onset and progression. Oral dysbiosis disrupts gastric microbiota through swallowing, bloodstream, or immune-mediated pathways, leading to mucosal barrier dysfunction and chronic inflammation. Translocation of microbes like P. gingivalis promotes gastric atrophy and IM (17). Reduced oral microbial diversity in saliva, subgingival, and buccal sites correlates with gastric precancerous lesions, serving as potential biomarkers for CAG (17). This article explores how oral microbes drive gastric microbiome shifts and metabolite production via the oral-gut axis, offering new insights for precision prevention and treatment. A comprehensive summary of etiological, immunological, and microbiome-related mechanisms is presented in **Supplementary Table 1**.

## Etiology and pathogenesis: beyond *Helicobacter pylori*

A 2024 study (1) analyzing 734,892 observational samples reported a 39.4% prevalence of atrophic gastritis in *H. pylori*-positive individuals, significantly higher than in negatives, particularly in high gastric cancer incidence areas. The pathogenesis of CAG extends beyond *H. pylori* infection, involving multifactorial synergies. Although *H. pylori* is considered the primary pathogen, it is not exclusive (18). *H.–pylori* induces chronic inflammation by activating transforming growth factor-beta 1 (TGF-β1) and connective tissue growth factor pathways, leading to mucosal atrophy and IM. Specifically, the oncogenic CagA protein is directly translocated into gastric epithelial cells via the bacterial type IV secretion system (T4SS) encoded by the cag pathogenicity island (cagPAI). This T4SS forms a pilus-like structure that acts as a molecular syringe, injecting CagA as a monomeric protein across the host cell membrane (19–21). Strain heterogeneity further modulates oncogenic potential, with CagA-positive strains—particularly those carrying multiple EPIYA tyrosine-phosphorylation motifs (EPIYA-A, B, C in Western strains; A, B, D in East Asian strains)—significantly elevating gastric cancer (GC) risk. Strains with ≥2 EPIYA-C repeats or the East Asian EPIYA-D motif exhibit the strongest oncogenic signaling (21–23). These strains drive enhanced IL-1β and TNF-α secretion, hypochlorhydria, and genomic instability—including impaired DNA repair, activation of mutation-inducing AID, and aberrant Wnt/β-catenin signaling. A key consequence is microsatellite instability (MSI), defined as insertion/deletion mutations in repetitive DNA sequences due to defective mismatch repair (e.g., downregulation of MLH1/MSH2), a recognized carcinogenic pathway in *H. pylori*-associated gastric cancer. Not all infected individuals progress equally, as host polymorphisms in cytokine genes interact with bacterial genotypes to drive differential inflammation and mutagenesis rates. For example, carriers of the pro-inflammatory IL1B-511T allele and the IL1RN2 allele are associated with increased risk of gastric precancerous lesions and gastric cancer, particularly in non-Asian populations; conversely, the IL1B-31C allele is linked to reduced risk in Asian populations, while the TNFA-308 G/A polymorphism shows no consistent association with gastric precancerous lesions or cancer risk (24). This variability underscores the need for genotype-specific risk stratification in *H. pylori*-associated CAG. Studies show *H. pylori* infection correlates with elevated IL-17A, IL-21, and IL-23 levels, which play key roles in gastric inflammation (25–27). For instance, IL-17 is markedly increased in *H. pylori*-infected gastric mucosa, associated with MAPK/ERK1/2 pathway activation (28, 29). Additionally, *H. pylori* influences TGF-β signaling to promote gastric inflammation (30). This infectious CAG typically manifests as antral-predominant atrophy, transitioning from hyper- to hypoacidity, fostering bacterial overgrowth.

Critiquing evidence heterogeneity is essential. Observational and mechanistic studies on H. pylori-CAG associations diverge. Epidemiological cohorts support H. pylori eradication in reducing CAG risk; multiple observational studies identify H. pylori as a major gastric cancer risk factor, with successful eradication linked to decreased CAG and cancer risk (31, 32). However, meta-analyses of mechanistic and observational studies (33, 34) highlight non-H. pylori factors, such as environmental toxins (e.g., nitrates, salt intake, smoking) and genetic susceptibilities (e.g., IL-1β polymorphisms), as crucial drivers of persistent gastric precancerous pathology even post-eradication. This heterogeneity stems from design differences: cross-sectional studies overlook longitudinal dynamics, while randomized controlled trials (RCTs) are often limited to infectious subtypes. A study (35) on preoperative upper gastrointestinal endoscopy in obese patients showed chronic gastritis (without H. pylori) as the most common type (35.2%), followed by H. pylori-related (20.1%), with autoimmune gastritis at 0.3%. Thus, phenotype-based subclassification is advocated: infectious CAG emphasizes antibiotic eradication and vaccine development; autoimmune CAG requires immunomodulatory therapies like vitamin supplementation and biologics (e.g., anti-IL-17 inhibitors). This subtyping optimizes strategies, avoiding one-size-fits-all approaches.

Chronic atrophic gastritis comprises two major etiologic subtypes with markedly different geographic and ethnic distributions (36). H. pylori seroprevalence exceeds 50% in high-risk Asian regions (e.g., East and Southeast Asia) and developing areas like Africa and Latin America, where it drives ~25% overall CAG prevalence and elevates risk 2.4-fold in infected individuals (2, 37, 38). This pattern persists in Asian immigrant communities in Europe and North America, reflecting sustained transmission. In contrast, Western countries with declining H. pylori prevalence (<20–30% in Northern Europe and North America) show lower overall CAG rates, where autoimmune CAG—though relatively rare (0.5–2% globally)—is more frequently identified, particularly among individuals of European ancestry (2, 37, 38). The two subtypes are largely mutually exclusive: active H. pylori infection suppresses parietal-cell autoantibodies and autoimmune gastritis via Th2 immune responses, while autoimmune CAG patients are nearly universally H. pylori-negative due to extensive mucosal destruction rendering the environment uninhabitable. This geographic–ethnic integration underscores the necessity of region- and ancestry-specific screening and management algorithms.

Environmental factors like diet (high-salt, high-nitrate) and smoking amplify these mechanisms. High-salt diets enhance H. pylori virulence and induce DNA methylation aberrations, silencing tumor suppressors (35). Bile reflux, often exacerbated by hepatolithiasis (intrahepatic bile duct stones), represents another critical environmental cofactor in CAG progression, particularly in Western cohorts where cholesterol or pigment stones predominate following cholecystectomy or biliary interventions. This condition promotes chronic mucosal inflammation through direct bile acid irritation and bacterial carriage, fostering dysbiosis and neoplastic risk independent of H. pylori (39). In high-prevalence Eastern regions, hepatolithiasis further amplifies geographic disparities in CAG burden via recurrent cholangitis and bile-mediated carcinogenesis. Overall, a pathogenesis perspective beyond H. pylori emphasizes multifactorial models, including interactive networks of inflammation, immunity, and metabolism. This explains H. pylori-negative CAG etiology and paves the way for personalized interventions. Building upon this foundational understanding of CAG pathophysiology, the subsequent sections delineate the clinical implications of microbiome dysbiosis and immune dysregulation within the oral-gut axis, tracing how these molecular and microbial perturbations manifest in disease progression and inform targeted diagnostics and therapies.

## Pathophysiological mechanisms and clinical implications: the oral-gut axis in CAG

Microbiota dysbiosis is a reversible risk factor in CAG, particularly within the oral-gut axis framework. This axis underscores how oral microbes influence gastrointestinal ecology via swallowing, blood, or lymphatic routes, amplifying systemic inflammation. Emerging evidence links oral microbial dynamics to gastric atrophy and IM, with the oral-gut axis promoting microbial translocation and functional shifts in CAG patients (14). This section examines three interconnected modules—oral microecology (including various measurable oral indicators), gastrointestinal microbiota-related indicators, and the oral-gut axis—using contemporary molecular profiling techniques, predominantly 16S rRNA gene sequencing, shotgun metagenomics, and targeted/untargeted metabolomics. Although fully integrated multi-omics analyses that simultaneously combine several omics layers remain scarce in this field, studies employing one or more of these approaches consistently demonstrate that oral and gastric microbial shifts drive profound metabolic reprogramming in CAG: reduced short-chain fatty acids, bile acid dysregulation, elevated TMAO and branched-chain amino acid derivatives, and subsequent activation of pro-inflammatory and oncogenic pathways (40). These alterations contribute to glandular atrophy, IM, and extragastric manifestations including type 2 diabetes, cardiovascular disease, and neuroinflammation in both *H. pylori*-related and autoimmune CAG (41, 42).

## Oral microecology: including various measurable indicators in the oral cavity

The oral microecosystem is a critical starting point for CAG pathogenesis, extending beyond salivary microbiota to include periodontal pockets, dental plaque, tongue coating, buccal mucosa, and gingival tissues (43, 44). These regions’ microbial diversity is closely tied to CAG onset and progression. The oral microbiome exhibits high diversity, including anaerobes like P. gingivalis and Fusobacterium nucleatum, associated with upper gastrointestinal dysfunction and promoting gastric inflammation (43, 45, 46). In CAG patients, oral dysbiosis manifests as increased periodontitis, with pathogens translocating to the stomach, disrupting acid secretion and altering gastric microbial composition (47). For example, P. gingivalis induces autophagy inhibition, leading to SCFAs metabolic disorders and exacerbating gut barrier damage (48). Studies show reduced Neisseria, Staphylococcus, and Hemophilus in CAG patients’ oral microbiota, with increased oral-derived microbes colonizing the stomach, promoting precancerous changes (49, 50). Oral dysbiosis also correlates with upper gastrointestinal precancerous lesions, with compositional changes in saliva, subgingival, and buccal microbiota reflecting gastric mucosal inflammation. Further evidence indicates that oral microbial dynamics are closely linked to gastric atrophy and IM, emphasizing the early role of oral microecology in CAG progression (51). To elucidate the oral microbiome’s role in CAG, specific measurable indicators in these niches provide key insights into disease mechanisms and diagnostic potential.

### Periodontal pockets

These are common sites for periodontitis, with microbial indicators showing increased abundance of anaerobes like P. gingivalis and Tannerella forsythia (52, 53). These bacteria can enter the stomach via swallowing, promoting *H. pylori* colonization or aggravating mucosal inflammation, driving CAG progression. A recent meta-analysis (54) showed that *H. pylori* infection increases the risk of periodontitis by more than 2-fold, supporting a bidirectional oral–gastric colonization pathway. Mechanistically, P. gingivalis gingipains and LPS–TLR4 signaling amplify IL-6/IL-17-driven inflammation, creating a pro-inflammatory niche that sustains *H. pylori* persistence and accelerates gastric atrophy (55). Consistently, higher loads of these periodontopathic bacteria in dental plaque, together with frequent detection of *H. pylori* in periodontal pockets of CAG patients, correlate with deeper pockets and more severe gastric pathology (56). Studies reveal higher *H. pylori* positivity in CAG patients’ periodontal pockets, potentially inducing gastric atrophy indirectly via cytokines like IL-6 (57).

### Dental plaque

As a primary oral biofilm, dental plaque serves as an *H. pylori* reservoir linked to chronic gastritis. Co-aggregation of *H. pylori* with F. nucleatum in plaque facilitates gastric migration (58). CAG patients often exhibit increased *H. pylori* or similar pathogens in plaque, migrating via the oral-esophageal route to disrupt gastric acid secretion and mucosal barriers, accelerating atrophic changes.

### Tongue coating

Microbial indicators here include fungi like *Candida albicans* and bacteria like Veillonella parvula. CAG patients show reduced tongue coating diversity with elevated *H. pylori* colonization (59, 60). Notably, *H. pylori* detection rates in tongue coating reach 20%–50%, continuously stimulating gastric mucosa via salivary swallowing, potentially accelerating glandular atrophy through cytokines like IL-8 and tumor necrosis factor-alpha (TNF-α) (61, 62). Research links *H. pylori* in tongue coating to metabolomic markers like elevated sphinganine 1-phosphate and rothia mucilaginosa et. al, suggesting microbial infection regulates host lipid metabolism reprogramming (63).

### Buccal mucosa

Key microbial indicators in the buccal mucosa include Lactobacillus spp. and Prevotella intermedia, which serve as markers of dysbiosis associated with increased risk of CAG progression, particularly in *H. pylori*-positive cases (19, 64). Studies (65, 66) have demonstrated elevated adherence of *H. pylori* to buccal epithelial cells in CAG patients, likely resulting from bidirectional systemic infection feedback loops, where oral colonization facilitates gastric reinfection or persistence (56). These microbes trigger local inflammation in the oral cavity, which indirectly influences the gastric mucosa through systemic immune responses (65). For instance, they promote alterations in secretory IgA (sIgA) levels, characterized by reduced sIgA production in saliva and mucosal surfaces, impairing barrier function and allowing enhanced microbial translocation via the oral-gut axis (40, 49). These sIgA changes exacerbate gastric mucosal vulnerability by diminishing local antimicrobial defenses, thereby amplifying autoimmune components of CAG, such as anti-parietal cell antibody production and Th17-mediated inflammation (67).

### Gingival tissue

Indicators primarily include Aggregatibacter actinomycetemc-omitans and periodontitis-causing bacteria. CAG patients show elevated inflammatory microbiota (e.g., Treponema denticola) in gingiva. T. denticola and A. actinomycetemcomitans produce toxins (e.g., LPS), with high abundance linked to periodontitis and systemic inflammation, potentially exacerbating CAG inflammation and atrophy via the oral-gut axis (68).

## Gastrointestinal tract: microbiota-related indicators

Gastric and intestinal microbial shifts are prominent in CAG. While *H. pylori* is a key gastric pathogen, environmental changes like reduced acidity (hypochlorhydria) permit oral bacterial colonization of gastric mucosa, enriching non-*H. pylori* taxa like Streptococcus and Lactobacillus while depleting beneficial Bifidobacterium (35, 69). This shift induces persistent inflammation via TLR4 activation, promoting mucosal atrophy and IM progression (70). In CAG patients, gastric microbial diversity decreases with elevated Firmicutes abundance, while gut dysbiosis reduces SCFAs (e.g., butyrate), amplifying inflammation. Studies indicate *H. pylori* infection alters gastric composition, causing bile acid dysregulation and advancing carcinogenic cascades (71). Metagenomic analyses show reduced gastric mucosal microbial abundance and complexity in CAG, but increased Firmicutes colonization correlates with chronic inflammation. Gut microbial changes also relate to CAG progression, with post-*H. pylori* eradication dysbiosis potentially persisting, affecting anticancer immunity and treatment efficacy.

## Oral-gut axis

Dysbiosis of the oral-gut axis contributes to systemic disorders, including cardiovascular and autoimmune diseases, by allowing microbial products and metabolites to enter the bloodstream. Key mediators include TMAO, a microbiota-derived metabolite, and LPS, an endotoxin released by Gram-negative bacteria. These factors trigger NF-κB activation and oxidative stress, thereby amplifying systemic inflammation and disease progression (72–74). Serum and mucosal metabolites change significantly in *H. pylori*-related gastritis, involving amino acid, lipid, and bile acid dysregulations (75). The axis connects oral and gastrointestinal microbiota, amplifying CAG progression through translocation and metabolic interactions (76). Oral bacteria like P. gingivalis enter the stomach via swallowing, altering gastric composition, promoting bile acid accumulation and β-catenin activation, leading to IM (77). Systemic impacts include elevated TMAO exacerbating vascular inflammation and influencing neuroinflammation via the gut-brain axis (78, 79). Studies show the oral-gut axis affects intestinal barriers and inflammation through microbial translocation, with TMAO as a key metabolite promoting atherosclerosis and systemic inflammation (80, 81).

Beyond gastrointestinal pathology, *H. pylori*’s extragastric implications are increasingly recognized, extending to metabolic disorders such as type 2 diabetes through chronic inflammation and insulin resistance modulation via cytokine dysregulation; cardiovascular conditions, where TMAO elevation correlates with atherosclerosis and endothelial dysfunction; and neurodegenerative diseases, including Alzheimer’s, potentially via neuroinflammatory pathways triggered by systemic microbial products like LPS crossing the blood-brain barrier. These associations underscore the multidisciplinary scope of *H. pylori* infection, where oral-gut axis perturbations contribute to a broader spectrum of chronic diseases, highlighting the need for integrated, cross-organ management approaches. Oral-gut axis dysbiosis also relates to aging and sarcopenia via chronic low-grade inflammation and mitochondrial dysfunction, aggravating CAG’s systemic effects.

Therapeutic strategies targeting the oral–gut axis are gaining traction. Berberine-containing herbal capsules restore microbial balance, inhibit P. gingivalis growth, and regulate SCFAs and bile acid metabolism by modulating interferon-gamma (IFN-γ) and TGF-β pathways, improving metabolic profiles (82). Clinical trials show probiotic supplementation (e.g., Lactobacillus) reduces CAG inflammatory markers and enhances oral hygiene and metabolic profiles (83). Nevertheless, the current evidence base remains limited by a paucity of large-scale, RCTs; most studies are observational or small interventional cohorts, underscoring the need for robust longitudinal data to establish causality and long-term efficacy. Overall, the oral-gut axis offers novel targets like oral hygiene interventions combined with FMT, potentially reversing CAG progression by restoring microbial and metabolic balance to interrupt carcinogenic cascades (84). These evolving clinical strategies highlight the importance of early, accurate detection and risk stratification, which form the focus of the following section on advanced multi-omics and non-invasive diagnostic approaches.

## Inflammation and immune responses: drivers of progression

Building on the etiological subtypes and pathophysiological foundations outlined earlier, this section delineates the immune dysregulation central to CAG progression, emphasizing distinctions between infectious and autoimmune forms. Chronic inflammation is the core driver of CAG progression to IM and gastric cancer (85), particularly in the Correa cascade, evolving from *H. pylori*-induced inflammation to atrophy, metaplasia, and dysplasia. This inflammatory microenvironment amplifies epithelial damage and immune dysregulation, closely linked to the oral-gut axis. Key cytokines like prostaglandin E2 (PGE2) and epidermal growth factor (EGF) dominate, activating the phosphatidylinositol 3-kinase/protein kinase B (PI3K/AKT) pathway to promote epithelial proliferation, inhibit apoptosis, and induce barrier dysfunction (86, 87). Specifically, PGE2 activates downstream EP receptors (e.g., EP2/EP4), enhancing NF-κB transcriptional activity and overexpressing pro-inflammatory cytokines like IL-1β and IL-6, disrupting tight junction proteins (e.g., ZO-1, occludin) and increasing mucosal permeability (88). EGF, via EGFR transactivation of PI3K/AKT, promotes cell survival and migration but chronic exposure leads to DNA damage accumulation and elevated cancer risk, synergizing with metabolomic changes (e.g., lipid dysregulation) to amplify systemic inflammation (89). These mechanisms extend beyond the stomach via the oral-gut axis: oral microbes such as P. gingivalis translocate and release microbial products, including LPS from the outer membrane of Gram-negative bacteria, which further activate PI3K/AKT, reinforcing inflammatory cascades and disrupting metabolic balance, such as reduced SCFAs and elevated TMAO (17). Pylori-induced inflammation is predominantly Th1-driven, involving IFN-γ and TNF-α secretion, which disrupts tight junction proteins (e.g., claudin-4), increasing permeability and promoting bacterial influx for sustained inflammation cycles. *H. pylori*’s CagA injects into host cells, activating MAPK and NF-κB pathways, inducing oxidative stress and CXCR2-mediated senescence—a process driven by the chemokine receptor CXCR2 that promotes cellular senescence primarily in gastric epithelial cells and neutrophils, leading to persistent inflammation, tissue remodeling, and inhibition of cell proliferation, driving atrophy and IM progression (90–92).

In *H. pylori*-positive CAG, this Th1 response amplifies microbial dysbiosis, with oral-derived bacteria like F. nucleatum synergizing via swallowing to recruit neutrophils and generate reactive oxygen species (ROS), exacerbating mucosal damage. In contrast, autoimmune CAG infiltration features CD4+ T cells and plasma cells producing anti-parietal cell antibodies targeting H+/K+-ATPase, causing hypoacidity and glandular atrophy. Plasma cell-secreted IgG and IgA antibodies activate complement and Fc receptor-mediated inflammation, linked to vitamin B12 deficiency and iron dysregulation (93, 94). This autoimmune mechanism predominates in *H. pylori*-negative patients, interacting with oral dysbiosis: changes in tongue coating or gingival microbiota amplify systemic autoimmune responses via IgA fluctuations (95).

Immune dysregulation is pivotal in CAG. Basic research reveals *H. pylori* suppresses regulatory T cells (Tregs) via CagA, amplifying Th17 responses: CagA disrupts Foxp3 expression, reducing Treg suppression and leading to IL-17 oversecretion, promoting neutrophil recruitment and ROS production (96–98). IL-17 activates downstream STAT3, inducing pro-inflammatory cycles and amplifying oxidative stress via mitochondrial damage, further promoting ferroptosis and mucosal atrophy (99). The oral-gut axis is key here: microbes from periodontal pockets or dental plaque in the oral cavity translocate to the gastric mucosa or enter the systemic circulation, where they amplify Th17 responses and sustain systemic inflammation.

## Diagnosis and management: toward precision strategies

**Figure 2:**
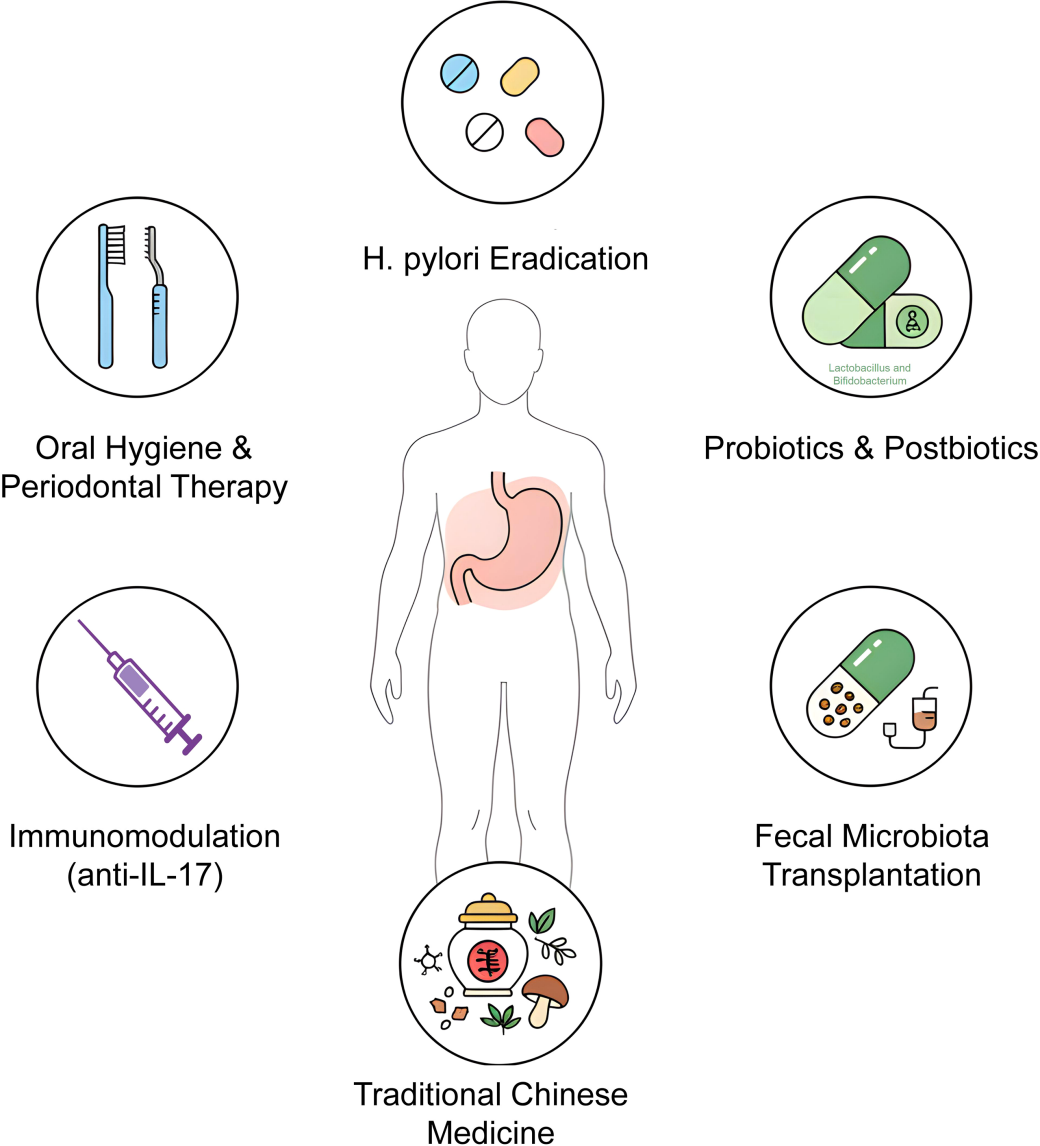
Integrated precision interventions targeting the oral-gut axis in chronic atrophic gastritis. Six interventions are depicted in a symmetrical layout: (1) oral hygiene and periodontal therapy to reduce periodontopathogen translocation; (2) *Helicobacter pylori* eradication therapy (e.g., bismuth-containing quadruple regimen); (3) probiotics and postbiotics (e.g., *Lactobacillus reuteri*, Bifidobacterium); (4) fecal microbiota transplantation (FMT) for comprehensive microbiome reconstruction; (5) traditional Chinese medicine compounds (e.g., berberine, Hericium erinaceus); and (6) targeted immunomodulation (e.g., anti-IL-17 monoclonal antibodies, primarily for autoimmune CAG).

To move beyond *H. pylori*-centered paradigms, a multidimensional precision strategy targeting the entire oral-gut axis is required (**Figure 2**). This integrated framework combines oral microbial control, pathogen eradication, microbiome reconstruction, herbal compounds, and targeted immunomodulation to achieve synergistic restoration of gastric mucosal homeostasis.

Endoscopy is the cornerstone for assessing gastric mucosal atrophy and IM (100). High-definition endoscopy combined with narrow-band imaging (NBI) or blue laser imaging significantly enhances detection of subtle lesions (101), particularly in high-incidence regions like Asia. Flexible spectral imaging color enhancement improves contrast between neoplastic lesions and surrounding mucosa, addressing limitations of traditional white-light endoscopy and boosting early gastric cancer identification (102). However, endoscopy’s invasiveness and subjectivity limit universality, overlooking early oral-gut axis signals. Non-invasive serological markers like pepsinogen (PG) levels address this: PG I/II ratio <3.0 indicates atrophy risk, combined with Operative Link for Gastritis Assessment (OLGA) staging to quantify severity, with OLGA III/IV increasing gastric cancer risk 5–10-fold (103, 104). Despite these advances, diagnostic modalities must account for clinical complexities, particularly in vulnerable populations. In older adults, age-related physiological changes such as immunosenescence and gastric mucosal atrophy can diminish the sensitivity of traditional tests; for instance, urea breath tests and serological assays exhibit reduced accuracy (sensitivity dropping to 70–80%) due to hypochlorhydria and altered antibody responses, necessitating confirmatory endoscopy or alternative non-invasive options. Novel molecular diagnostics, including stool antigen tests—which offer >90% specificity for active *H. pylori* infection—and salivary 16S rRNA sequencing for oral microbiota profiling, provide complementary tools for early detection and monitoring, especially in resource-limited settings or for tracking post-treatment persistence.

Oral microecological indicators offer new perspectives for integrated diagnosis. Elevated P. gingivalis in periodontal pockets, detectable via salivary 16S rRNA sequencing, serves as a non-invasive biomarker for CAG, reflecting oral-gut axis microbial translocation and inflammation-driven processes (105). Metabolomics reveals serum and urine amino acid and lipid dysregulations with 85% diagnostic accuracy, complementing histological assessments (40). Nomogram models based on serum CXCL5 levels optimize risk stratification in obese or elderly populations, integrating oral microbial data (e.g., tongue coating *H. pylori* colonization) for cross-organ comprehensive diagnosis (106). In older adults, immunosenescence and gastric mucosal aging exacerbate *H. pylori* persistence, reducing diagnostic accuracy (e.g., urea breath test sensitivity) and increasing eradication failure due to comorbidities and polypharmacy (107). Age-related decline in adaptive immunity and innate immunity impairs clearance of oral periodontopathogens and gastric colonizers, fostering persistent low-grade systemic inflammation driven by elevated IL-6, TNF-α, and microbial products that sustain gastric Th1/Th17 responses and accelerate glandular atrophy and IM (108, 109). Concurrently, oral-gut dysbiosis exacerbates immunosenescence through chronic antigen stimulation, molecular mimicry, and altered vitamin D metabolism (110), creating a vicious cycle that is particularly detrimental in autoimmune CAG and in age-related alveolar bone loss seen in severe periodontitis—conditions that share common immunosenescent mechanisms including dysregulated M1 macrophage polarization, accumulation of senescent B/T cells, and energy metabolic reprogramming. Tailored strategies, including probiotics and susceptibility-guided regimens, are essential to mitigate these challenges and improve outcomes in this high-burden demographic. This age-specific lens highlights the interplay between immunosenescence and oral-gut dysbiosis in accelerating CAG progression.

Integrated management focuses on oral-gut axis interventions, transcending sole *H. pylori* eradication to emphasize multidimensional, synergistic strategies. Quadruple therapy (proton pump inhibitor, bismuth, and two antibiotics) achieves >90% eradication rates, reversing early atrophy and reducing gastric cancer risk by 20%–30% (111, 112). However, post-eradication patients persist with microbiota dysbiosis, leading to reduced SCFAs and secondary bile acid accumulation, or maintaining inflammation and cancer risk via Wnt/β-catenin (113, 114). Probiotics (e.g., *Lactobacillus reuteri*) and postbiotics restore oral-gut balance, reducing pathogen translocation like P. gingivalis and improving SCFAs metabolism (115). Periodontal therapies (e.g., ultrasonic scaling) lower inflammatory microbiota in pockets and plaque (e.g., F. nucleatum), decreasing TMAO production and alleviating systemic inflammation’s impact on gastric mucosa (116, 117). FMT reconstructs oral-gut microbiota ecology, significantly reducing inflammatory markers (e.g., IL-6, TNF-α), especially effective in *H. pylori*-negative CAG (118).

Fusion of Eastern and Western management paradigms is crucial. Eastern strategies combine *H. pylori* eradication with herbal interventions (e.g., Hericium erinaceus modulating NF-κB to inhibit oral-derived inflammation), while Western emphasizes autoimmune CAG immunomodulation, such as vitamin B12 supplementation and anti-IL-17 monoclonal antibodies (e.g., secukinumab) targeting Th17-mediated cascades. Integrated interventions—combining periodontal treatment, probiotics, and herbs—synergistically target oral and gastric microecology, interrupting Correa cascades (119–121). As these strategies evolve, future perspectives must prioritize longitudinal RCTs to validate long-term efficacy, explore axis-specific biomarkers for personalized prophylaxis, and address global disparities in access to susceptibility-guided therapies.

## Conclusion

In summary, CAG pathogenesis transcends traditional *H. pylori*-centric models, integrating microbiome dysbiosis, metabolic alterations, and immune dysregulation via the oral-gut axis. Microbial translocation from oral niches amplifies inflammation and carcinogenesis, with functional shifts in metabolites like SCFAs and TMAO driving progression. Key clinical messages include the utility of oral biomarkers for early risk stratification and the synergy of probiotics with eradication therapies to restore axis balance. Diagnostic evolution toward non-invasive biomarkers and multi-omics integration enables precision risk assessment, while management shifts to holistic interventions including probiotics, FMT, and cross-disciplinary therapies. Major knowledge gaps persist in causal validation of oral translocation and long-term post-eradication dysbiosis effects, particularly in aging populations. Future research should prioritize RCTs to validate these links, develop axis-targeted prophylactics, and conduct longitudinal multi-omics trials in high-burden regions, ultimately reducing global gastric cancer burden through personalized, axis-informed strategies.

## Data Availability

The original contributions presented in the study are included in the article/**Supplementary Material**. Further inquiries can be directed to the corresponding authors.
