# Supplementary material for: The oral-gut axis in chronic atrophic gastritis: current perspectives and integrated strategies
Source: Front Immunol. 2026 Jan 8;16:1699501. doi: 10.3389/fimmu.2025.1699501 (PMC12823898; doi:10.3389/fimmu.2025.1699501)
Supplement: Supplementary file 1 [file Table1.docx]

## **Supplementary Table 1**

## **Comprehensive Mechanistic and Clinical Summary of Chronic Atrophic Gastritis (CAG)**

| **Domain** | **Key Concepts** | **Mechanistic / Clinical Insights** |
| --- | --- | --- |
| **Etiology** | *Helicobacter pylori, Autoimmunity, Environmental Factors* | - *H. pylori* (particularly East Asian CagA EPIYA-D variant) induces IL‑1β, TNF‑α, NF‑κB, and Wnt/β‑catenin activation.- Autoimmune CAG involves anti-parietal cell antibodies, intrinsic factor deficiency, and B12 malabsorption.- High salt diet, bile reflux, smoking, and nitrosamines contribute to mucosal damage. |
| **Pathogenesis** | *Chronic Inflammation, Immunometabolic Remodeling* | - Persistent Th1/Th17-driven inflammation promotes epithelial atrophy.- MAPK, PI3K/AKT, STAT3, and β‑catenin pathways are dysregulated.- SCFAs decrease while secondary bile acids and TMAO increase, promoting oxidative stress and mucosal injury. |
| **Oral–Gastric Axis** | *Oral Microbial Translocation & Inflammatory Amplification* | - Oral taxa (e.g., *Porphyromonas gingivalis*, *Fusobacterium nucleatum*) colonize gastric mucosa.- Translocated oral microbes disrupt autophagy and tighten junctions, impairing mucosal defense.- Tongue coating, dental plaque, gingival sulcus, and buccal mucosa serve as microbial reservoirs. |
| **Gastrointestinal Microbiome** | *Dysbiosis in Stomach & Intestine* | - *H. pylori*–positive stomach shows decreased diversity with increased *Streptococcus* and *Lactobacillus*.- Intestinal SCFAs decline, perpetuating pro-inflammatory signaling.- Overgrowth of Firmicutes is frequently observed. |
| **Immune Mechanisms** | *Th1/Th17 Dominance, Autoantibodies* | - Th1 cytokines (IFN‑γ, TNF‑α) and Th17 cytokines (IL‑17, IL‑23) drive epithelial apoptosis and atrophy.- Autoimmune CAG features complement-mediated destruction of parietal cells.- IL‑17–STAT3 axis increases ROS production and accelerates intestinal metaplasia. |
| **Clinical Subtypes** | *Infectious vs Autoimmune* | - Infectious CAG: predominates in Asia/Africa, involves antral atrophy.- Autoimmune CAG: more common in Western populations, characterized by body/fundic gland atrophy and hypochlorhydria. |
| **Diagnostics** | *Endoscopy, Serology, Microbiome, Multi-Omics* | - Image-enhanced endoscopy (NBI/BLI/FICE) improves detection of early metaplastic lesions.- Serum PG I/II ratio <3 suggests high-risk atrophic changes (OLGA III–IV).- Oral microbiome signatures (e.g., *P. gingivalis*) may serve as early risk markers.- Metabolomic profiling increases diagnostic accuracy (~85%). |
| **Elderly-Specific Considerations** | *Immunosenescence, Diagnostic Challenges* | - Urea breath test and serological sensitivity decrease (70–80%).- Aging correlates with impaired microbial clearance and chronic low-grade inflammation.- Higher susceptibility to mucosal injury and rapid progression. |
| **Management Strategies** | *Multidimensional Interventions* | - Bismuth-containing quadruple therapy remains first-line for *H. pylori* eradication (>90% success).- Post-eradication dysbiosis requires adjunctive microbiota-directed therapy (probiotics, postbiotics).- Periodontal therapy reduces oral pathogenic load and systemic inflammation.- Fecal microbiota transplantation (FMT) shows potential for restoring oral–gut microbial homeostasis.- Phytochemicals (e.g., berberine, Hericium erinaceus) modulate NF‑κB and improve metabolic balance. |
| **Systemic Impacts** | *Cardiometabolic & Neurological Links* | - Associated with insulin resistance, atherosclerosis (via TMAO), and neuroinflammation mediated by LPS and cytokines. |
| **Knowledge Gaps & Future Directions** | *Causality, Precision Medicine* | - Need for large-scale longitudinal multi-omics studies.- Development of oral–gastric axis biomarkers and targeted vaccines.- Exploration of personalized microbiota modulation strategies. |
